# Alcohol consumption among pregnant women in James Town Community, Accra, Ghana

**DOI:** 10.1186/s12978-017-0384-4

**Published:** 2017-09-26

**Authors:** Joanita Da Pilma Lekettey, Phyllis Dako-Gyeke, Samuel Agyei Agyemang, Moses Aikins

**Affiliations:** 1 Greater Accra Regional Hospital, Accra, Ghana; 2 Department of Social and Behavioral Sciences, School of Public Health, College of Health Sciences, University of Ghana, Legon, Accra, Ghana; 3 Department of Health Policy, Planning and Management, School of Public Health, College of Health Sciences, University of Ghana, P. O. Box LG13, Legon, Accra, Ghana

**Keywords:** Alcohol consumption, Alcoholic beverage, Alcohol users, Pregnant women, Ghana

## Abstract

**Background:**

Alcohol consumption among pregnant women is a public health concern, considering its adverse outcomes for both mother and the developing foetus. This study examined factors that facilitate prenatal alcohol consumption, knowledge of adverse outcomes of prenatal alcohol exposure and alcohol expenditure among pregnant women in an urban community in Ghana.

**Methods:**

In June 2014, a survey was conducted among 250 pregnant women sampled from James Town, an urban community in the Greater Accra Region of Ghana. Data were collected through face-to-face interviews and descriptive statistics conducted. The prevalence of alcohol consumption among women was determined. Pearson chi-square was used to determine associations between variables where necessary.

**Results:**

Fifty-four percent of the pregnant women were aged 20 – 29 years. Seventy-three percent reported that they have ever consumed an alcoholic beverage before pregnancy. Of these, 77% take alcohol “once a while” and 48% reported taking alcohol during pregnancy. Most of the pregnant women (53%) who currently consume alcoholic beverages had it from friends, and their main reason for prenatal alcohol consumption was socialization (39%). Majority of both current alcohol drinkers (78%) and non-current alcohol drinkers (74%) were aware that prenatal alcohol consumption can lead to spontaneous abortion. Additionally, current alcohol drinkers spend averagely GHS 4.54 (SD 4.63) on their favourite alcoholic drink and overall, also spend averagely GHS 4.63 (SD 4.82) on their entire alcoholic beverage weekly. Over two-thirds (63%) of women reported monthly average income of less than GHS200.

**Conclusion:**

This study shows high prenatal alcohol consumption in James Town, Accra, despite pregnant women’s knowledge of its adverse effects on the developing foetus. Alcohol is usually sourced from friends with socialization noted as a major reason for prenatal alcohol consumption. These results could be used to inform future health advocacies and policies on prenatal alcohol exposure and maternal and child health interventions in the country.

## Plain English Summary

The consumption of alcohol during pregnancy is recognized as harmful and a global health problem. This practice is known to predispose the unborn child to premature delivery, low birth weight and fetal alcohol syndrome. We study pregnant women in James Town, Accra, Ghana because of its populated settings, high fertility rate among the youth of this population, as well as its cosmopolitan nature. Health literacy where pregnant women have the capacity to obtain, process, and understand basic health information and services needed to make appropriate health decision may be an important factor in understanding the harmful use of alcohol during pregnancy.

Cross-sectional data were analysed. A sample size of 250 pregnant women were involved, more than half of the respondents were unmarried (68.4%) and 60.4% of the women were petty traders.

The findings revealed that almost half of women consume alcohol during pregnancy, the main source for their alcoholic beverage is through friends and majority of the pregnant women are aware that prenatal alcohol consumption can lead to spontaneous abortion, still birth, low birth weight and mental disability.

In conclusion, though majority of respondents who drank alcohol knew the harmful effects of prenatal alcohol consumption, they continued to drink during pregnancy. The challenge is to develop effective health promotion messages to reach women of child-bearing age so they can make informed decisions regarding alcohol consumption during pregnancy.

## Background

Alcohol consumption during pregnancy has been a public health concern, considering its possible adverse outcomes for both mother and developing foetus [1–4]. Prenatal alcohol consumption comprises the drinking of any alcoholic beverage during pregnancy or before pregnancy is noticed [5]; that is the entire period within which a mother carries an embryo through its developmental stages [6]. Although this stage is often characterised by several unfavourable health conditions (e.g. acid reflux, constipation, nausea and vomiting among other symptoms) there is caution against the use of some substances, including alcohol [6]. Studies have observed the possible harmful effects of alcohol on pregnancy, including premature delivery, structural malformations, pre- and post-natal growth retardation, central nervous system damage, hyperactivity and attention problems, increased risk of sudden infant death syndrome, low birth weight, and learning and memory deficits [5, 7–10]. A study found that exposure to heavy drinking (over 48–60 g. ethanol/day) may cause fetal alcohol syndrome [11]. For maternal health, studies have found a relationship between alcohol consumption and spontaneous abortion [5, 7, 8]. It is worthy to note that whereas some of these outcomes may be reversible through interventions, others may not.

The need to address prenatal alcohol consumption has become key considering the observed increasing trend of harmful alcohol consumption among women. The WHO Global Disease Burden Report (2015) indicated that from the year 2000 to 2012, there was about a 30% increase in deaths from alcohol use disorders among females [2]. This increase observed among women is double the overall increase (15%), and much higher than increases reported among men (12%). A literature review in France found the prevalence of alcohol use during pregnancy ranged from 12% to 63% [12], and 22% in Netherlands [13]. Recent studies conducted among sub-Saharan African (SSA) women show a similar pattern of increasing rates of alcohol consumption [1, 3].

The Ghana Demographic and Health Survey estimates the median age at first birth among women age 25-49 at 21.4 years [14]. In Ghana, Asamoah and Agardh (2012) reported a positive association between alcohol consumption and the risk of maternal deaths from induced abortion [7]. Unfortunately, abortion has been identified as one of the leading direct obstetric causes of the rather high maternal mortality rates in Ghana [15].

The far-reaching effects of prenatal alcohol consumption call for global and country specific policy interventions to curb harmful alcohol use during pregnancy. In 2008, the first national alcohol policy for Ghana was drafted. Following this, the government has put in a lot of effort to control alcohol consumption and to reduce the harm to high-risk populations [16]. The ability to develop relevant policies that can be implemented across the country requires a better understanding of prenatal alcohol consumption patterns across Ghana. Although another study suggests the prevalence of maternal alcohol use among rural populations in Ghana [17], much is not known about urban populations. The general objective of the study was to assess alcohol consumption among pregnant women in an urban community, James Town, specifically to determine prevalence, knowledge on effects of alcohol in pregnancy, reasons and factors associated with prenatal alcohol ingestion and types of alcoholic beverages consumed by pregnant women in James Town.

## Methods

### Study design and population

This was a quantitative and cross-sectional study conducted among pregnant women in James Town, an urban community in central Accra. James Town is located within Asheidu Keteke Sub metro in the Greater Accra Region, Ghana. Ashiedu Keteke Sub Metro which is located at the centre of Accra Metropolitan Assembly is the smallest yet the most populated in the Accra Metropolis. The population of the Sub Metro is a mixture of traditional indigenous Gas and migrant population from various parts of the country. The major occupation within the traditional areas is fishing. Their women are mostly fish mongers and petty traders [18]. James Town is one of the communities in Asheidu Keteke with neighbourhoods such as Pilla Enyo, Palladium day care and Kofi Oku. The major health problems in Ashiedu Keteke include; poor environmental sanitation, overcrowding, as well as other common diseases [19]. Ussher Polyclinic, Princess Marie Louise Hospital (PML), Makola Clinic, James Town Maternity, Beach Clinic, Ghana Post Clinic, Fire Medical Centre and Judicial Clinic provide public health care services to the people of Ashiedu Keteke and beyond. In addition, there are thirteen private health facilities which also supplement health care services to the populace. The study population are women within the reproductive age group (18-49 years) who are pregnant and living within the James Town Community. The total population of James Town Community is estimated to be 15,771 [20] and total expected pregnancy is 4% of the total population. A study in the UK showed that the density of youth-orientated bars and clubs influence the volume of alcohol intake especially among the youth [21]. The setting was selected for the study because of its cosmopolitan nature with a lot of small drinking bars and spots.

### Sample size determination and sampling

The estimated population of pregnant women in James Town is about 630.Cochran [22] estimates the sample size from the population as follows:

$$ \boldsymbol{n}=\frac{{\boldsymbol{z}}^{\mathbf{2}}\boldsymbol{p}\left(\boldsymbol{1}\boldsymbol{\hbox{-}}\boldsymbol{p}\right)}{{\boldsymbol{d}}^{\boldsymbol{2}}} $$

Where.

n = sample size.

z = the confidence interval.

p = prevalence rate.

d = margin of error.

Assuming an expected prevalence rate of 50% to be able to detect statistically significant association within a 5% margin of error at 95% confidence interval, the sample size, n was determined.

$$ {\displaystyle \begin{array}{l}\boldsymbol{n}=\frac{\mathbf{1.}{\mathbf{96}}^{\mathbf{2}}\boldsymbol{x}\mathbf{0.5}\left(\mathbf{1}-\mathbf{0.5}\right)}{\mathbf{0.}{\mathbf{05}}^{\mathbf{2}}}\hfill \\ {}\mathbf{n}=\mathbf{384.12}\hfill \end{array}} $$

Therefore using the finite population correction factor with the formula given as:

$$ {\displaystyle \begin{array}{l}\frac{n}{1+\frac{n}{N}}\hfill \\ {}\frac{384}{\left(1+\frac{384}{630}\right)}\hfill \\ {}=238.5\hfill \end{array}} $$

Approximated to 250.

Simple random sampling was used to sample the pregnant women. We randomly selected one neighbourhood in the community (*Pilla Enyo*) as the starting point. A pen was tossed and the direction of the head of the pen was used to select the first house. After a pregnant woman in a selected house is identified, she consents and is interviewed. Pregnancy was confirmed by observation and through word of mouth by sampled women themselves. Pregnant women were obtained in the following neighbourhoods in James Town - *Pilla Enyo*, *Kofi Oku*, *Binney Villa*, *Keta House* and*James Town Maternity* area.

### Data collection

The fieldwork was done within a period of two weeks (7th June, 2014 to 21st June, 2014). Structured questionnaires were administered to pregnant women who consented. A total of 256 questionnaires were administered, out of which 250 were valid representing a response rate of 97.66%. The questionnaire covered the socio-demographic characteristics, alcohol consumption, duration of pregnancy, frequency of alcohol consumption, types of alcohol consumed, facilitators of alcohol consumption, knowledge of adverse effect of parental alcohol exposure and cost of alcoholic beverages. A structured questionnaire was used for data collection. It consisted of closed ended questions. The structured questionnaire was pretested on a sample of 20 pregnant women to find out difficulties in understanding the meaning of the questions and to estimate the amount of time to answer all the questions. Alcohol consumed was not measured in this study. Respondents were asked on average the number of tots or bottles they consumed per day. A tot of an alcoholic beverage is equivalent to 30mls. A bottle of alcoholic beer could be either 350mls or 500mls of an alcoholic beverage. However, the questionnaire did not indicate if the bottle of alcohol consumed was 350mls or 500mls. Face-to-face interviews were conducted in the popular local language (*Ga)*, and translated back into English. Interviews took an average of 25 min.

### Data analysis

Descriptive statistics were conducted to determine prevalence of alcohol consumption among women. The knowledge on the adverse effects of prenatal alcohol exposure and expenditure on alcohol and food were analysed according to current alcohol users and non-current alcohol users. Data entry and analysis were done using Epi Info Version 7 and Microsoft excel. Pearson chi square was used to determine associations between expenditure on food by alcohol and non-alcohol drinkers.

## Results

### Socio-demographic characteristics of pregnant women

Majority 134 (54%) of the respondents were between 20 and 29 years, unmarried 171 (70%), petty traders 151 (60%) and those who had attained Junior Secondary Education 100 (40%). Eighty-four percent of the respondents were Christians and over two-thirds 123 (63%) of them earned monthly average income of less than GHS200. Overall 120 (48%) of the pregnant women indicated consuming alcoholic beverage during their current pregnancy. While 51 (42.5%) of pregnant women in age group 18 years or less indicated prenatal alcohol consumption, 5 (4.2%) of the pregnant women who are 35 years and above admitted consuming alcohol in their current pregnancy. Prevalence of prenatal alcohol consumption among pregnant women with no formal education was 22 (18.3%) and 9 (7.5%) among pregnant women with secondary or tertiary education. While the entire traditionalist group indicated prenatal alcohol consumption, 96 (80%) and 7 (5.8%) of pregnant women who consumed alcohol were Christians and Muslims respectively (Table 1).

**Table 1:** Socio-demographic characteristics of pregnant women

Characteristics	Number (%)	Current alcohol users (%)
	*N* = 250	*N* = 120
Age (years):		
< 19	56 (22.4)	51(42.5)
20 – 24	66 (26.4)	37(30.8)
25 – 29	68 (27.2)	16(13.3)
30 – 35	36 (14.4)	11(9.2)
35+	24 (9.6)	5(4.2)
Marital status:		
Married	79 (31.6)	37(30.8)
Unmarried	171 (68.4)	83(69.2)
Education:		
No education	32 (12.8)	22(18.3)
Primary	91 (36.4)	49(40.8)
Junior High School	100 (40.0)	40(33.3)
Senior High School/Vocational/Tertiary	27 (10.8)	9(7.5)
Occupation:		
Petty Trader	151 (60.4)	77(64.2)
Artisan	33 (13.2)	16(13.3)
Civil Servant	7 (2.8)	2(1.7)
Unemployed	53 (21.2)	20(16.7)
Other	6 (2.4)	5(4.2)
Religion:		
Christian	210 (84.0)	96(80)
Muslim	21 (8.4)	7(5.8)
Traditionalist	12 (4.8)	12(10)
No religion	7 (2.8)	5(4.2)
Income^a^ (GHS)		
< 200	123 (63.1)	63(66.3)
> 200	72 (36.9)	32(33.7)

^a^Only 195 pregnant women reported income

### Alcohol consumption among pregnant women

About 183 (73%) of the pregnant women were reported to have ever consumed alcoholic beverage before pregnancy. The average number of bottles of alcoholic beverage consumed per day was 1.2 (SD 0.7) while the average number of tots consumed per day was 1.4 (SD 0.5). Table 2 shows that pregnant women consumed alcohol “once a while” (77%) whilst others consumed alcohol weekly (14%) and daily 17 (9%). Almost half 120 (48%) reported taking alcohol during pregnancy. Of these, 65 (54%) consumed alcohol once a while, others consumed it weekly (38%) and some (8%) daily. Most pregnant women consumed Guinness or beer 72 (60%), *pito* 18 (15%), spirits/gin/bitters 13 (11%), palm wine/other wines 9 (8%) and *Akpeteshie (*7%). Most of the pregnant women were in their third trimesters 54 (45%) whilst 17 (14.2%) and 49 (40.8%) were in their first and second trimesters respectively.

**Table 2:** Frequency and type of alcoholic beverage consumed

Frequency of consumption	Number (%)
Before pregnancy:	(*n* = 183)
Daily	17 (9.3)
Weekly	25 (13.7)
Once a while	141 (77.0)
During pregnancy:	(*n* = 120)
Daily	10 (8.3)
Weekly	45 (37.5)
Once a while	65 (54.2)
Type of alcoholic beverage:	(*n* = 120)
Guinness/Beer	72 (60.0)
Spirits/Gin/Bitters	13 (10.8)
*Akpeteshie* ^a^	8 (6.7)
Palm wine/other wine	9 (7.5)
*Pito* ^a^	18 (15.0)
Pregnancy timing	(*n* = 120)
First Trimester	17 (14.2)
Second Trimester	49 (40.8)
Third Trimester	54 (45.0)

^a^Locally manufactured alcoholic beverages

### Facilitators of alcohol consumption among pregnant women

Most of the pregnant women 97 (53%) who currently consume alcoholic beverage sourced it from friends. Over 62 (33%) sourced it themselves whilst 20 (11%) indicated that their spouse or boyfriends bought it for them. For majority of the pregnant women, 65 (55%) affirmed they consume alcoholic beverage at home, about 31 (26%) consume at drinking spots, 13 (11%) consume at naming ceremonies and 7 (6%) at funeral ceremonies.

### Reasons for alcohol consumption among pregnant women

Table 3 shows the main reasons for pre-natal alcohol consumption among pregnant women included socialization/excited/way of life 47 (39%), and aided in managing life pressure/stress/unemployment 18 (15%). More women 42 (35%) also chose to drink because they needed it to boost their appetite for food. Some 6 (5%) pregnant women also indicated other reasons for drinking alcohol such as “takes when husband takes”, obey tradition, to prevent fever and healing of sores in stomach.

**Table 3:** Reasons for drinking alcohol during pregnancy

Reason	Number (%)
Life pressure/stress/unemployment/bored/“kill time”	18 (15.1)
Easily available/accessible	7 (5.8)
Socialize/excited/way of life	47 (39.2)
Appetizer	42 (35.0)
Others^a^	6 (5.0)

^a^Others include “takes when husband takes”, obey tradition, to prevent fever and healing of sores in stomach

### Perceived adverse effects of prenatal alcohol consumption

**Fig. 1:**
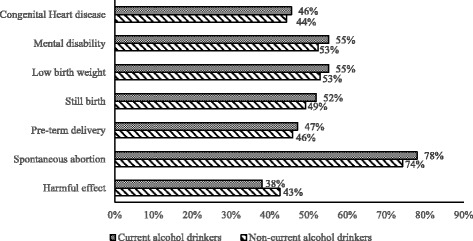
Perceived adverse effects of pre-natal alcohol consumption

Majority of the pregnant women, both current alcohol drinkers 143 (78%) and non-current alcohol drinkers 50 (74%) were aware that prenatal alcohol consumption can lead to spontaneous abortion. About 101 (55%) of current alcohol drinkers and 36 (53%) of non-current alcohol drinkers knew it causes low birth weight. Averagely, over 40% of both current alcohol drinkers and non-current alcohol drinkers knew prenatal alcohol consumption can cause congenital heart diseases, still birth or pre-term delivery. About 29 (43%) of non-current alcohol drinkers and 70 (38%) of current alcohol drinkers were aware of the harmful effect of prenatal alcohol consumption (Fig. 1).

### Cost of alcohol use during pregnancy

Non-current alcohol drinkers spent an average of GHS 11.16 (SD 8.08) on food compared to current alcohol drinkers (GHS 10.54; SD 11.00) (Table 4). The difference was statistically significant (*p* < 0.001). Additionally, current alcohol drinkers spent averagely GHS 4.54 (SD 4.63) on their favourite alcoholic drink weekly and overall, spent averagely GHS 4.63 (SD 4.82) on their entire alcoholic beverage consumption.

**Table 4:** Average weekly expenditure on food and alcohol among pregnant women

Items	Average expenditure (GHS)	
	Non-current alcohol users (*N* = 67)	Current alcohol users (*N* = 183)
Meal/food (min, max), SD	11.16 (1.50,40.00), ± 8.08	10.54 (1.50,70.00), ± 11.00
Favorite alcoholic drink (min, max), SD	–	4.54 (0.50, 30.00), ± 4.63
Entire alcoholic drink (min, max), SD	–	4.63 (0.50, 32.00), ± 4.82

## Discussion

In this study we report prevalence of alcohol consumption, pregnant women’s level of knowledge on prenatal alcohol consumption, reasons why pregnant women drink alcoholic beverage and types of alcoholic beverages consumed. We found out that almost half of women consumed alcohol during pregnancy. This is higher as compared to a cross-sectional study by Adusi-Poku et al., (2012) in Ghana in the Bosomtwi District of the Ashanti Region which estimated the prevalence of prenatal alcohol consumption to be 20.4% [17]. Also, the Ghana Demographic and Health Survey reports 18% of women in Ghana drink alcoholic beverage [15]. This difference is not surprising as the difference could be attributed to contextual differences. Also, Adusi-Poku’s study was facility based whereas this study was community based. Probably, women recruited through facilities are very conscious than those recruited through the community. Another reason could be that those recruited through the facility may not be biased in their responses.

The findings of this study also revealed that most of those who consumed alcohol were mostly young and less educated. This is contrary to findings from a study in Netherlands where older women and those with higher education consumed more alcohol [13]. A recent study in France found that moderate drinking during pregnancy was associated with higher educational level [23]. The Ghana Demographic and Health Survey also reports a relatively young median age of 21.4 years at first birth among women age 25-49 [14].

Findings from this study suggest that pregnant women although might be aware of the possible dangers of alcohol use in pregnancy have not adjusted their behavior accordingly. Those who consumed alcohol during pregnancy (current alcohol users) consumed averagely more than one bottle or tot of alcohol per day. The findings of this study are not different from a Ghanaian study where study participants consumed on average half to one tot of alcohol during a sitting session [24]. Results in this study showed majority of the pregnant women drank beer either occasionally or frequently. A similar cross-sectional study on prevalence and predictors of Maternal Alcohol Consumption in 2 Regions of Ukraine reported that 92.7% of pregnant women surveyed had consumed alcohol before, 54.8% reported drinking alcohol in the first month of the pregnancy and 46.3% continued to drink alcohol throughout pregnancy [25]. Another study in the Netherlands found that, 69% of women consumed alcohol within 6 months before pregnancy and 22% during pregnancy [13]. This study found that among women, 73.2% had previous history of consuming alcohol. Prevalence of prenatal alcohol consumption exists despite the advice from World Health Organization, and the British Medical Association (BMA) that cautions pregnant women to refrain from alcohol, particularly during the first trimester [26, 27]. Traditionally, the primary industry of occupation for the people of Ga Mashie has been fishing. Historically, the dynamics of the industry has always called for division of labor between men (who are involved in the actual fishing activities) and women (who have been mainly responsible for preservation, marketing and trading). Hence most of these women in the coastal town are financially independent themselves. Also, due to the cosmopolitan nature of this study site, the people are known to be involved in lots of social events. Hence, the prevalence of alcohol is likely to be high in this setting compared to the national average. This was very evident during the data collection where drinking spots were found in most homes entered with alcoholic beverages openly available for sale.

The alcohol content of beer is about 4%. One would think this would be expensive considering the fact that 60.4% are petty traders and 21.2% are unemployed. However in James Town, where outdoorings (naming ceremony), funerals and parties are very frequent, these pregnant women can have access to beer if they attend such events. Over 8% of pregnant women who currently consume alcohol admitted to drinking alcoholic beverage on a daily basis. About a quarter of those who consumed alcohol preferred Pito (a locally brewed alcoholic beverage) probably due to the fact that some homes produced the alcoholic beverage locally and was readily available and cheap. Wine was the least preferred alcoholic beverage in our findings. The study’s findings are not consistent with the study by Adusi-Poku et al., (2013) who reported that the preferred alcoholic beverage that majority (36.4%) of the pregnant women consumed was akpeteshie (a locally brewed alcoholic beverage), liqueurs 27.3%, Guinness 18% and Beer 3.9%, which in this finding was the most preferred [24]. Personal preferences might have influenced the observed differences.

This study has shown that the main source for their alcoholic beverage is through friends. More women also chose to consume alcohol because they needed it to boost their appetite for food. While most of these women preferred to drink at home, others go to drinking spots or naming ceremonies. Eaton et al., (2012) reported that 61.0% of the pregnant women favoured attending the bar to drink [28]. The findings also indicated that non-current alcohol consumers were likely to spend more on food compared to alcohol consumers. This may be because, current alcohol consumers also spend extra money on alcoholic beverage. A survey by Slow Food UK on students in some universities in the UK found that the average student weekly expenditure on alcohol is £20 or more, whilst around £10 is spent on food [29].

Furthermore, our study revealed most (52%) of the pregnant women (current and non-current drinkers) knew the harmful effect of prenatal alcohol consumption yet consumed. This practice is indicative of addictive behaviour and also disregard for consequences considering the alcohol consumed might be free. For instance, majority of the pregnant women were aware that prenatal alcohol consumption can lead to spontaneous abortion, stillbirth, low birth weight and mental disability. A study in the late 90’s in Western Cape on alcohol consumption by pregnant women estimated that 59.7% of pregnant women were aware that alcohol could have harmful effects on the foetus whilst 35.6% of the significant drinkers had a grade of insight or knowledge, ranging between minimal to excellent knowledge of the possible effects of alcohol on pregnancy and 8% of the women who apparently did not drink alcohol indicated any insight in this regard [30]. One of the limitations of our study is that pregnancy was by observation but not hospital confirmation. Another limitation is that prenatal alcohol consumption was self-reported. Unfortunately pregnant women may under report or over report prenatal alcohol usage.

## Conclusion

In summary, this study found that most pregnant women had previous history of consuming alcohol, however, it is encouraging that many women who drank alcohol reduced their alcohol intake during pregnancy. The linkage of prenatal alcohol consumption to fetal or infant development has several important policy and programmatic implications. Specifically, the fact that majority of women who consumed alcohol knew the harmful effects of prenatal alcohol consumption, yet continued to consume during pregnancy calls for promoting early literacy programs and ensuring basic needs are met to reduce the risk of problems during pregnancy and ensure the infant’s health and development. To further improve problems associated with alcohol use during pregnancy, women need access to comprehensive health care to address their unmet health needs before and throughout their pregnancy. This should be followed by public health strategies to help them make informed decisions about reducing their alcohol consumption or abstaining. In addition, antenatal clinics can make screening for alcohol ingestion among attendees a part of protocols.

## Abbreviations

BMA: British Medical Association
GDHS: Ghana Demographic and Health Survey
SSA: Sub Saharan Africa
WHO: World Health Organisation

## References

[1] Culley CL, Ramsey TD, Mugyenyi G, Kiwanuka GN, Ngonzi J, Macleod S (2013). Alcohol exposure among pregnant women in sub-saharan Africa: a systematic review. J Popul Ther Clin Pharmacol.

[2] GBD 2015 Mortality and Causes of Death Collaborators (2016). Global, regional, and national life expectancy, all-cause mortality, and cause-specific mortality for 249 causes of death, 1980-2015: a systematic analysis for the Global Burden of Disease Study 2015. Lancet.

[3] Martinez P, Roislien J, Naidoo N, Clausen T (2011). Alcohol abstinence and drinking among African women: data from the World Health Surveys. BMC Public Health.

[4] United Nations (2016). The sustainable development goals report.

[5] Bradley KA (1998). Badrinath S, Bush K, Boyd-Wickizer, Anawalt B. Medical risks for women who drink alcohol. J Gen Intern Med.

[6] WHO (2016). Health Topics: Pregnancy.

[7] Asamoah BO, Agardh A (2012). Alcohol consumption in relation to maternal deaths from induced-abortions in Ghana. Reprod Health.

[8] Ethen MK, Ramadhani TA, Scheuerle AE, Canfield MA, Wyszynski DF, Druschel CM (2009). Alcohol consumption by women before and during pregnancy. Matern Child Health J.

[9] Jacobson JL, Jacobson SW (2002). Effects of prenatal alcohol exposure on child development. Alcohol Res Health.

[10] Meyer-Leu Y, Lemola S, Daeppen JB, Deriaz O, Gerber S (2011). Association of moderate alcohol use and binge drinking during pregnancy with neonatal health. Alcohol Clin Exp Res.

[11] Ornoy A, Ergaz Z (2010). Alcohol Abuse in Pregnant Women: Effects on the Fetus and Newborn, Mode of Action and Maternal Treatment. Int J Environ Res Public Health.

[12] Dumas A, Simmat-Durand L, Lejeune C (2014). Pregnancy and substance use in France: a literature review. J Gynecol Obstet Biol Reprod.

[13] Lanting CI, van Dommelen P, van der Pal-de Bruin KM, Bennebroek Gravenhorst J, van Wouwe JP (2015). Prevalence and pattern of alcohol consumption during pregnancy in the Netherlands. BMC Public Health.

[14] Ghana Statistical Service, Ghana Health Service, ICF Macro (2014). Ghana demographic and health survey 2014.

[15] Ghana Statistical Service, Ghana Health Service, ICF Macro (2009). Ghana demographic and health survey 2008.

[16] Modern Ghana. National alcohol draft policy launched. [http://www.modernghana.com/news/255087/1/national-alcohol-draft-policy-launched.html]. Accessed 23 Aug 2016.

[17] Adusi-Poku Y, Edusei AK, Bonney AA, Tagbor H, Nakua E, Otupiri E (2012). Pregnant women and alcohol use in the Bosomtwe district of the Ashanti region-Ghana. Afr J Reprod Health.

[18] Mahama AS, Acheampong AT, Peprah OB, Boafo YA (2011). Preliminary Report for Ga Mashie Urban Design Lab, Spring 2011.

[19] Ashiedu Keteke Sub Metro - Accra Metropolitan Assembly. Annual Report 2013. Accra: Accra Metropolitan Assembly; 2014.

[20] Ghana Statistical Service, Ghana Health Service, ICF Macro (2010). Ghana demographic and health survey 2009.

[21] Roberts M (2015). ‘A big night out’: Young people’s drinking, social practice and spatial experience in the ‘liminoid’ zones of English night-time cities. Urban Stud.

[22] Cochran WG. Sampling techniques. 2 edition. New York: Wiley; 1963.

[23] Dumas A, Toutain S, Simmat-Durand L (2017). Alcohol use during pregnancy or breastfeeding: A national survey in France. J Womens Health (Larchmt).

[24] Adusi-Poku Y, Bonney AA, Antwi GD (2013). Where, When and What Type of Alcohol Do Pregnant Women Drink?. Ghana Medical Journal.

[25] Chambers CD, Yevtushok L, Zymak-Zakutnya N, Korzhynskyy Y, Ostapchuk L, Akhmedzhanova D (2014). Prevalence and predictors of maternal alcohol consumption in 2 regions of Ukraine. Alcohol Clin Exp Res.

[26] British Medical Association (BMA). Fetal Alcohol Spectrum Disorders - A Guide for Healthcare Professionals. London: British Medical Association; 2007.

[27] WHO (2006). Framework for Alcohol Policy in WHO European Region.

[28] Eaton LA, Kalichman SC, Sikkema KJ, Skinner D, Watt MH, Pieterse D (2012). Pregnancy, alcohol intake, and intimate partner violence among men and women attending drinking establishments in a Cape Town, South Africa township. J Community Health.

[29] Slow Food UK. Average British student spends more on alcohol than food. [https://www.slowfood.org.uk/average-british-student-spends-more-on-alcohol-than-food/]. Accessed 15 Nov 2016.

[30] Croxford J, Viljoen D (1999). Alcohol consumption by pregnant women in the Western Cape. S Afr Med J.

